# Among older African Americans, high‐risk ABCA7‐80 allele carriers have lower medial temporal lobe dynamic network flexibility than APOE‐ε4 allele carriers

**DOI:** 10.1002/alz.093970

**Published:** 2025-01-09

**Authors:** Miray Budak, Bernadette A. Fausto, Wiktoria Piaszczynska, Payton G. White, Mark A. Gluck

**Affiliations:** ^1^ Rutgers University–Newark, Newark, NJ USA

## Abstract

**Background:**

Alzheimer’s disease (AD) is a progressive neurodegenerative disease characterized by amyloid plaques and neurofibrillary tangles which cause neurodegeneration and cognitive decline1. APOE‐ε4 is identified as the first common genetic risk factor with high penetrance in the early onset of AD3. ABCA7 (rs115550680) (ABCA7‐80) is associated with the development of late‐onset AD among African Americans2. The medial temporal lobe (MTL) is the first region that displays atrophy and neurofibrillary tangle involvement during AD4. However, the comparative influence of APOE‐ε4 and ABCA7‐80 on neural functions among African Americans is not yet known. The present study investigated the effect of APOE‐ε4 and ABCA7‐80 high‐risk alleles on the MTL dynamic network flexibility among cognitively unimpaired older African Americans.

**Method:**

175 older, cognitively unimpaired African Americans drawn from the ongoing longitudinal study, Pathways to Healthy Aging in African Americans completed saliva collection for genotyping and underwent an MRI scan in a Siemens 3T Prisma scanner. AFNI and ANTs were used for dynamic functional MRI processing and analysis for MTL dynamic network flexibility. Gender, age, and education were used as covariates for analysis.

**Result:**

The analysis revealed trend‐level differences in MTL Dynamic network flexibility between high‐risk and non‐risk allele carriers for ABCA7‐80 (F = 3.531, η² = 0.025, p = 0.062) and for APOE‐ε4 allele (F = 2.421, η² = 0.017, p = 0.122). Individuals with the ABCA7‐80 high‐risk allele exhibited significantly lower MTL dynamic network flexibility compared to those with the APOE‐ε4 allele (F = 4.577, η² = 0.067, p = 0.036).

**Conclusion:**

ABCA7‐80 high‐risk allele may better capture the dynamic neural dysfunction in the MTL network rather than APOE‐ε4 among cognitively unimpaired older African Americans.

